# Reduced gut microbial diversity characterized by decreased *Bacteroides* is associated with vasovagal syncope in children

**DOI:** 10.3389/fcimb.2026.1764992

**Published:** 2026-04-14

**Authors:** Wei Tang, Qing Yang, Jiayu Ren, Xingyu Han, Cuifen Zhao

**Affiliations:** 1 Department of Pediatrics, Qilu Hospital of Shandong University, Jinan, China; 2 Department of Pediatrics, Women’s Hospital of Nanjing Medical University, Nanjing Women and Children’s Healthcare Hospital, Nanjing, China

**Keywords:** autonomic nervous system, gut microbiota, orthostatic intolerance, SCFAs, vasovagal syncope

## Abstract

**Background:**

Vasovagal syncope (VVS) represents the most prevalent form of orthostatic intolerance in children and is characterized by recurrent fainting episodes that substantially impair quality of life and psychosomatic well-being. The pathogenesis of VVS is primarily associated with dysregulation of the autonomic nervous system. Emerging evidence suggests that the gut microbiota, a complex microbial community residing in the human intestine, plays a pivotal pathological role in various autonomic nervous system related disorders. However, data on the gut microbiota profile in pediatric VVS remain limited. The present study therefore aims to characterize gut microbiota changes in children with VVS and its subtypes, providing novel insights into the underlying mechanisms and potential therapeutic strategies.

**Method:**

Fecal samples were obtained from 40 children diagnosed with VVS, comprising 20 with the vasodepressor subtype, 8 with the cardioinhibitory subtype, and 12 with the mixed subtype, as well as from 60 age-matched healthy controls. Microbial community composition was assessed through 16S rRNA gene sequencing. Alpha and beta diversity analyses were conducted to evaluate differences between the VVS and HC groups, as well as among the different VVS subtypes. Then, the bacterial flora with significant differences was identified based on microbial relative abundance data using the Wilcoxon rank-sum test, ANCOM-BC2, and LEfSe analyses.

**Results:**

Compared with HCs, children with VVS exhibited reduced alpha diversity, reflected by lower Simpson and Shannon indices. Beta diversity analysis indicated a modest shift in community structure based on Bray–Curtis and weighted UniFrac distances, accompanied by increased within-group dispersion in VVS. No significant differences were detected in dominant phyla. At the genus level, among the predominant taxa, ANCOM-BC2 identified decreased *Bacteroides* and increased *Prevotella_9* in the VVS group. LEfSe revealed reduced abundances of *Bacteroides* and *Faecalibacterium*. No significant differences in alpha or beta diversity indices were observed among the VVS subtypes including vasodepressor, cardioinhibitory, and mixed types.

**Conclusion:**

Our findings provide evidence of gut microbiota alterations in children with VVS and suggest that reduced microbial diversity, particularly the decreased abundance of the key genera *Bacteroides*, may be involved in the development of VVS.

## 1 Introduction

Orthostatic intolerance (OI) refers to a group of symptoms and signs caused by the body’s inability to tolerate prolonged upright posture. Vasovagal syncope (VVS) is the most common hemodynamic subtype of OI, characterized by transient loss of consciousness and muscle tone. often accompanied by presyncope symptoms such as dizziness, headache, chest tightness, fatigue, nausea, and vomiting (Tao et al., 2020). The onset of vasovagal syncope (VVS) is generally mediated by dysregulation of the autonomic nervous system (ANS) and typically resolves spontaneously following postural adjustment. Orthostatic intolerance (OI) is relatively common in the pediatric population, with an estimated one in six children experiencing at least one syncopal episode before adulthood (Shen et al., 2017). VVS has a high recurrence rate (Jorge et al., 2020), and limited responsiveness to pharmacological interventions (Sheldon et al., 2021). Over time, it not only affects children’s learning and quality of life but also causes serious harm to their physical and psychological health (Kara and Doğan, 2021). Consequently, elucidating the underlying mechanisms of VVS and developing effective therapeutic strategies are important research directions in the field of pediatrics.

The underlying pathophysiological mechanisms of VVS remain incompletely understood. Previous studies have implicated several contributing factors, including activation of the Bezold–Jarisch reflex (Longo et al., 2023), dysregulation of peripheral vascular tone (Wieling et al., 2016), disturbances in neurohumoral modulation (Stewart, 2012) and hemodynamic instability (Li et al., 2018). These processes are associated with disruption of the sympathetic–parasympathetic balance within the autonomic nervous system. With the rapid advancement of microbiome science, the gut microbiota residing within the human intestine has garnered increasing attention for its role in health and disease (Fan et al., 2023), Notably, it plays a critical part in regulating and sustaining the functional integrity of both the central and autonomic nervous systems (Naveed et al., 2021). Intestinal activity is modulated through the coordinated control of the ANS and the enteric nervous system (ENS). The ENS, a dense neuronal network embedded within the gastrointestinal wall, operates independently of the central nervous system (CNS) and is often referred to as the “second brain” (Schneider et al., 2019). The ENS and CNS are interconnected via a complex neuroendocrine signaling network known as the gut–brain axis (Dinan and Cryan, 2017). Recent studies have highlighted the pivotal role of the gut microbiota in the interaction along the brain-gut axis, together with the CNS and ENS forming the integrated brain–gut–microbiome (BGM) system. Normally, the BGM system maintains physiological homeostasis through bidirectional feedback via neuroendocrine signaling and immune responses, involving multiple signaling pathways such as the ENS, ANS and humoral factors. Any level of perturbations in this complex network can disrupt systemic equilibrium and contribute to disease onset (Dong and Mayer, 2024; Sah et al., 2024). These findings indicate that the gut microbiota participates in regulating human ANS function, and children with VVS typically present with symptoms indicative of ANS dysregulation (Tarbell et al., 2023). Based on this rationale, the present study uses 16S rRNA gene sequencing to compare the gut microbiota composition between children with VVS and healthy controls, as well as among different VVS subgroups, to identify characteristic microbial profiles associated with VVS. These findings aim to provide new insights and potential directions for the diagnosis and treatment of VVS in children.

## 2 Methods

### 2.1 Subject

Children diagnosed with VVS who presented with symptoms of OI at Qilu Hospital of Shandong University from July 2023 to July 2024 were enrolled as the case group. Healthy children undergoing routine physical examinations at the outpatient clinic were recruited as the control group during the same period. All participants underwent detailed medical history inquiries and physical examinations, followed by standardized fecal sample collection. In addition, all children in the case group completed a head-up tilt test.

The VVS group inclusion criteria were as follows: (1) Children aged 5–18 years who were diagnosed with VVS according to the 2018 Chinese Pediatric Cardiology Society (CPCS) guideline for diagnosis and treatment of syncope in children and adolescents and confirmed by the head-up tilt test; (2) Normal stool characteristics, defined as Bristol Stool Form Scale (BSFS) types III (like a sausage with cracks on its surface) and IV (like a smooth and soft sausage or snake); (3) Voluntary participation in the study with written informed consent obtained from the child or his/her legal guardian. The exclusion criteria were as follows: (1) Use of antibiotics, probiotics, or special diets within the past two months; (2) Presence of gastrointestinal symptoms such as diarrhea or constipation, or concurrent viral/bacterial infections within the past two months; (3) Known neurological, endocrine, or cardiovascular dysfunction or disease; (4) Improper or contaminated fecal sample collection.

The healthy children (HC group) inclusion criteria were as follows: (1) Healthy children aged 5–18 years with no history of OI-related symptoms; (2) Normal stool characteristics; (3) Voluntary participation with informed consent obtained from the child or guardian. The exclusion criteria were as same as the VVS group.

### 2.2 Head-up tilt test

Head-up tilt test (HUTT) (Deng et al., 2025) is currently the preferred method for diagnosing vasovagal syncope (VVS) and determining its subtypes. During the test, participants are tilted from a supine position to a predetermined angle to simulate the gravitational redistribution of blood that occurs during standing, thereby inducing neuro-mediated reflexes and presyncope or syncope symptoms. Based on simultaneous electrocardiogram and blood pressure monitoring, the hemodynamic type of VVS in each child can be determined.

To reduce the risk of vomiting or discomfort, participants fasted and abstained from drinking for at least 4 hours prior to testing. The test was conducted between 8:00 and 11:00 a.m. in a quiet, comfortable environment with soft lighting and a controlled temperature of 24 °C. Participants emptied their bladder and were positioned supine on an electric tilt table (Beijing Juchi Medical Technology Co., Ltd.) with the chest and knees secured, and connected to electrocardiogram and blood pressure monitoring devices. The head of the bed frame was then tilted upward to a 60° angle, and changes in heart rate, blood pressure, and ECG were recorded. The test was terminated immediately upon a positive response or after 45 minutes if no positive response occurred.

The positive criteria for HUTT were as follows (Wang et al., 2018): (1) Blood pressure decrease (systolic ≤80 mmHg, diastolic ≤50 mmHg, or mean arterial pressure reduction ≥25%); (2) Bradycardia (heart rate <75 bpm for children <6 years, <65 bpm for 7–8 years, <60 bpm for >8 years); (3) Sinus arrest or junctional escape rhythm; (4) Transient second-degree or higher atrioventricular block, or cardiac arrest lasting up to 3 seconds. The classification of VVS subtypes were defined as follows: the vasodepressor type (VT) is characterized predominantly by a decrease in blood pressure, the cardioinhibitory type (CT) by a predominant decrease in heart rate, and the mixed type (MT) by decreases in both blood pressure and heart rate.

### 2.3 Fecal sample collection

Prior to sample collection, participants were instructed to empty their bladders to minimize potential urinary contamination. All fecal samples were collected on site at the hospital under physician guidance following a standardized protocol. Fecal samples were self-collected into clean, dry containers under natural defecation conditions to avoid external contamination. Using sterile gloves, approximately 2 g of mid-portion feces was aseptically collected into a fecal DNA collection tube containing a DNA stabilizing reagent using a disposable sampling device (Jianshi Biotechnology Co., Ltd.). The tubes were then securely sealed. Immediately after collection, samples were placed in a cold-chain insulated container and transferred to the laboratory within 1 hour, and stored in a −80 °C ultra-low temperature freezer for microbiome sequencing analysis.

### 2.4 16S rRNA gene sequencing

Total genomic DNA was isolated from fecal samples using the QIAamp Fast DNA Stool Mini Kit (Qiagen, Germany) following the manufacturer’s protocol. The quality and integrity of the extracted DNA were assessed by 1% agarose gel electrophoresis, while DNA concentration and purity were measured using a NanoDrop 2000 spectrophotometer (Thermo Fisher Scientific, USA). Samples with an A260/A280 ratio between 1.8 and 2.0 were considered of acceptable purity. Each sample was tagged at the 5’ end of the primer with a unique barcode, and amplification was performed using the universal primers 341F (5’-CCTAYGGGRBGCASCAG-3’) and 806R (5’-GGACTACHVGGGTWTCTAAT-3’), targeting the V3–V4 hypervariable region of the bacterial 16S rRNA gene, with an expected amplicon length of approximately 470 bp. The PCR mixture contained 15 µL of Phusion High-Fidelity PCR Master Mix (New England Biolabs), 0.2 µM of each primer, and 10 ng of genomic DNA template. PCR amplification was performed with an initial denaturation at 98 °C for 1 min, followed by 30 cycles of denaturation at 98 °C for 10 s, annealing at 50–55 °C for 30 s, and extension at 72 °C for 30 s, with a final extension at 72 °C for 5 min. The reaction products were then held at 4 °C. Amplified PCR products were excised from agarose gels and purified using a DNA Gel Extraction Kit (Axygen, USA), followed by elution in TE buffer. The quality of the purified PCR products was assessed by 2% agarose gel electrophoresis, and DNA concentrations were quantified using a Qubit 4.0 fluorometer (Thermo Fisher Scientific, USA) and the Qubit dsDNA HS Assay Kit (Thermo Fisher Scientific, USA). PCR amplicons were pooled in proper ratios according to sequencing requirements, thoroughly mixed, and target bands were recovered based on electrophoresis results. A small-fragment library was then constructed according to the characteristics of the amplified V3–V4 region, and high-throughput sequencing of the 16S rRNA gene V3–V4 region was performed on the Illumina NovaSeq platform using a paired-end sequencing configuration of 2×250 bp.

All procedures were conducted in accordance with laboratory standard operating procedures (SOPs). Extraction blanks and PCR negative controls were included throughout the workflow. The absence of detectable amplification in negative controls was confirmed by agarose gel electrophoresis, indicating no evident exogenous DNA contamination. To minimize potential batch effects, DNA extraction, library preparation, and sequencing were performed in a randomized manner, with samples from the VVS and HC groups randomly distributed across processing batches.

### 2.5 Bioinformatic Analysis

#### 2.5.1 Read merging and preprocessing

The raw data generated from the Illumina platform were obtained in fastq format. Paired-end reads were assigned to their respective samples based on unique barcode sequences, after which barcode and primer sequences were trimmed and adapter sequences were removed. This demultiplexing and trimming process was performed using Cutadapt (version 3.3) implemented in Python (version 3.6.13). Subsequently, paired-end reads were merged using FLASH (version 1.2.11), and successfully merged sequences were retained as raw tags for downstream analyses.

#### 2.5.2 Quality filtering

Quality filtering of raw tags was performed using fastp (version 0.23.1). Sequences containing ambiguous bases, excessive homopolymers, or low-quality scores were removed according to the specific filtration criteria: (1) tags containing more than five ambiguous bases were discarded; (2) the quality threshold was set to 19, meaning bases with a quality score ≥19 were considered qualified; and (3) tags were removed if the proportion of unqualified bases exceeded 15%.

#### 2.5.3 Chimera removal

The clean tags were aligned against the SILVA reference database to identify potential chimeric sequences. Chimera detection and removal were performed using the vsearch package (version 2.16.0) in a reference-based mode. Sequences identified as chimeras were removed, and the remaining sequences were defined as effective tags for downstream OTU clustering and taxonomic analysis.

#### 2.5.4 OTU clustering

The effective tags were clustered into Operational Taxonomic Units (OTUs) at a 97% sequence similarity threshold using the UPARSE pipeline (v7.0.1001). During this process, singletons (sequences appearing only once) were removed to reduce noise and potential sequencing errors. For each OTU, the sequence with the highest frequency was selected as the representative sequence for further taxonomic annotation.

#### 2.5.5 Species annotation

Taxonomic annotation of representative OTU sequences was performed using the Mothur classification algorithm against the SILVA reference database (release 138.1). Based on the taxonomic annotation results and the OTU abundance table for each sample, taxonomic abundance profiles were generated at different taxonomic levels, including kingdom, phylum, class, order, family, genus, and species.

#### 2.5.6 Microbial community structure and diversity analysis

Alpha diversity indices, including ACE, Chao1, Simpson (1–*D*), and Shannon index, were calculated using QIIME software (v1.9.1). Beta diversity was evaluated and visualized using the *ade4* and *ggplot2* packages in R software (v4.0.3), based on Bray-Curtis and weighted UniFrac distance metrics. The significance of differences among groups was assessed using the Adonis test (PERMANOVA) and betadisper (PERMDISP) analysis implemented in the *vegan* packages in R. Microbial differential abundance analysis was conducted using a multi-step statistical framework. Phylum-level relative abundances were compared using Wilcoxon rank-sum tests with p-values adjusted using the false discovery rate (FDR) method. At finer taxonomic levels, differential abundance was assessed using ANCOM-BC2 (v2.12.0 in R v4.5.2), incorporating sample-specific bias correction, structural zero detection, and a 10% prevalence filter, with p-values adjusted by the Holm-Bonferroni method (*q* < 0.05). LEfSe (v1.0) was additionally applied for supplementary exploratory identification of discriminative taxa with a significance threshold set at an LDA score (log_10_) ≥ 4.

### 2.6 Statistical analysis

Normally distributed continuous variables were expressed as mean ± standard deviation (*X̅* ± *S*), with independent samples t-test employed for comparisons between two groups, and one-way ANOVA for comparisons among three groups. Non-normally distributed continuous variables were presented as median [lower quartile, upper quartile] (*M*[*Q*1, *Q*3]), the non-parametric test was used for the intergroup comparison between the two groups. and the Kruskal–Wallis H test was used for comparisons across three groups. Categorical variables were described as constituent ratios and compared using the chi-square test. A *P*-value < 0.05 was considered statistically significant.

## 3 Results

### 3.1 Demographic features

A total of 100 children were included in the study, comprising 60 healthy controls (HC) and 40 patients with vasovagal syncope (VVS), of whom 20 were classified as vasodepressor type (VT), 8 as cardioinhibitory type (CT), and 12 as mixed type (MT). All participants were Han Chinese, born and residing in Shandong Province, China, with comparable daily dietary habits and no specific dietary restrictions such as vegetarian or halal diets. There were no significant differences between the VVS and HC groups in terms of sex, age, weight, height, or body mass index (BMI) (*P* > 0.05) (**Table 1**).

**Table 1:** Basic characteristics of the study participants.

Items	VVS group (n=40)	HC group (n=60)	t/χ^²^	P
Boys/Girls, n	15/25	27/33	0.554	0.457
Age, years	11.06 ± 2.53	11.48 ± 2.75	0.789	0.432
Weight, Kg	45.60 ± 16.26	43.57 ± 13.14	-0.689	0.493
Height, cm	150.8 ± 14.01	150.98 ± 16.70	0.057	0.955
BMI, kg/m^2^	19.43 ± 4.40	18.66 ± 2.76	-0.982	0.33

### 3.2 Operational taxonomic units

**Figure 1:**
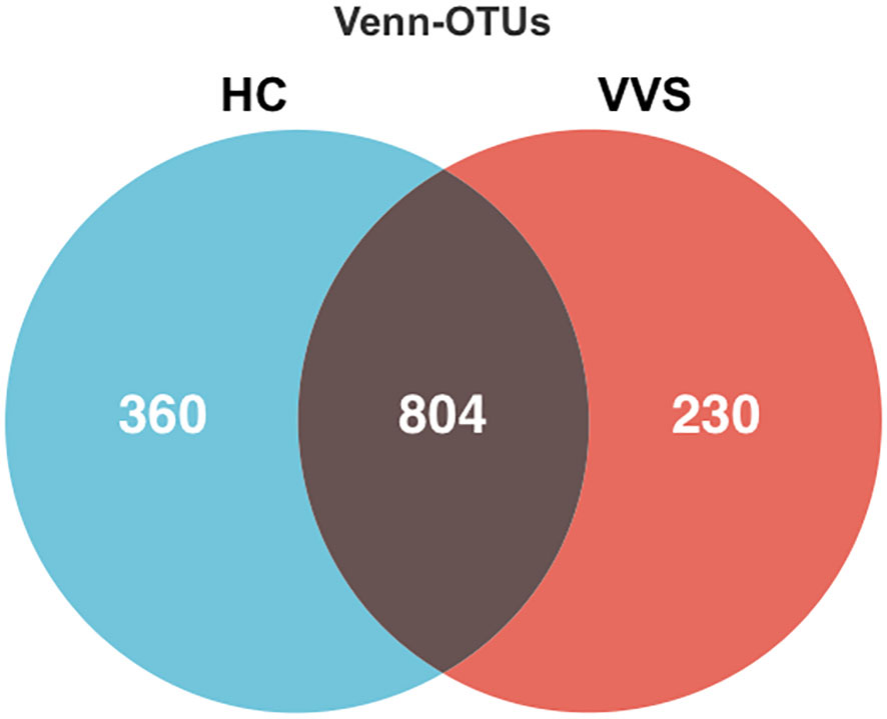
Veen diagram of gut microbiota between VVS and HC groups.

Fecal samples were sequenced using the Illumina NovaSeq platform. A total of 9,047,679 high-quality sequences were obtained from the 100 samples, with an average of 90,476 sequences per sample and an average sequence length ranging from 409.45 to 428.16 bp. OTUs were clustered at 97% sequence similarity, and a Venn diagram was used to illustrate the distribution and overlap of OTUs among groups. Among the 100 samples, 1,394 OTUs were identified in total. The VVS and HC groups contained 1,034 and 1,164 OTUs, respectively, with 804 OTUs shared between the groups, while 230 and 360 OTUs were unique to the VVS and HC groups, respectively. (**Figure 1**).

### 3.3 Alpha diversity analysis

Alpha diversity indices are applied to assess the richness and evenness of microbial communities among different groups. Specifically, the ACE and Chao1 indices estimate the number of observed and potential species within a sample, serving as indicators positively correlated with community richness. In contrast, the Shannon and Simpson indices take into account both species richness and evenness, thereby providing a comprehensive measure of overall community diversity. Higher values of these indices represent greater microbial diversity and a more complex community structure (Qian et al., 2020).

#### 3.3.1 VVS group vs HC group

**Figure 2:**
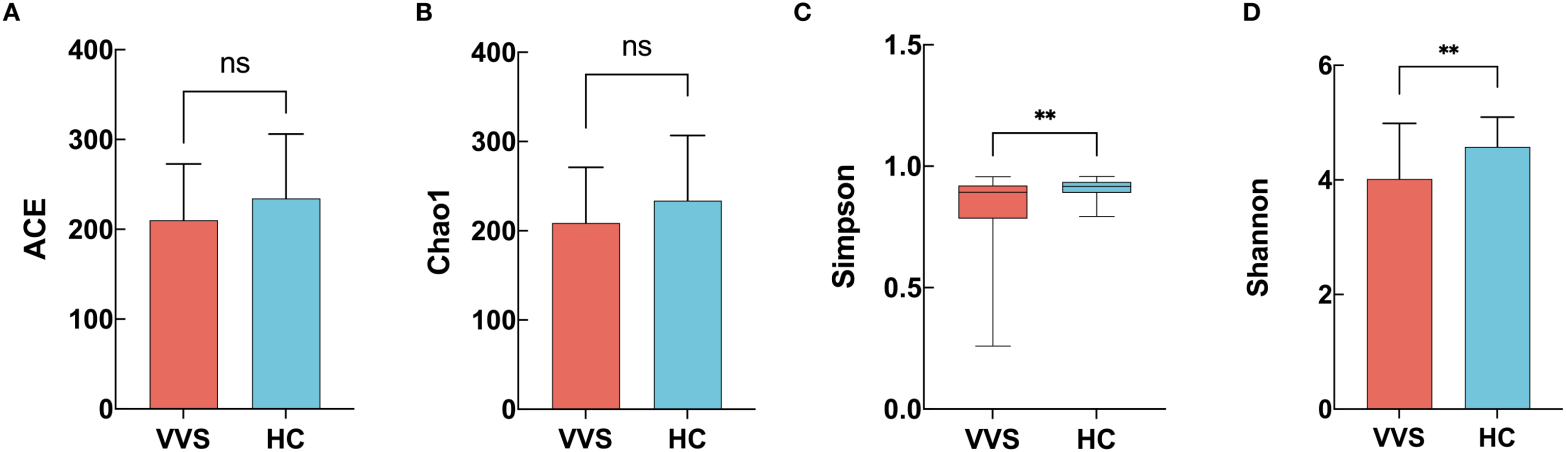
Comparison of gut microbiota alpha diversity indices between VVS and HC groups. **(A)** ACE index; **(B)** Chao1 index; **(C)** Simpson index; **(D)** Shannon index.

The comparison of ACE index showed no statistically significant difference between the VVS and HC groups (210.194 ± 62.478 vs 234.348 ± 71.681, *t* = 1.736, *P* = 0.086) (**Figure 2A**); Similarly, no significant difference in Chao1 index was observed between the VVS and HC groups (208.882 ± 62.439 vs 233.687 ± 72.983, *t* = 1.762, *P* = 0.081) (**Figure 2B**); In contrast, the Simpson index differed significantly between the VVS and HC groups (0.894[0.784,0.939] vs 0.916[0.891,0.936], *Z* = -3.040, *P* = 0.002) (**Figure 2C**), with lower values observed in the VVS group. Similarly, the Shannon index was significantly lower in the VVS group than in the HC group (4.016 ± 0.970 vs 4.579 ± 0.519, *t* = 3.365, *P* = 0.001) (**Figure 2D**). Taken together, the alpha diversity analysis revealed that children with VVS exhibited significantly reduced gut microbial diversity compared with healthy controls, primarily reflected by decreased community evenness.

#### 3.3.2 VT group vs CT group vs MT group

**Figure 3:**
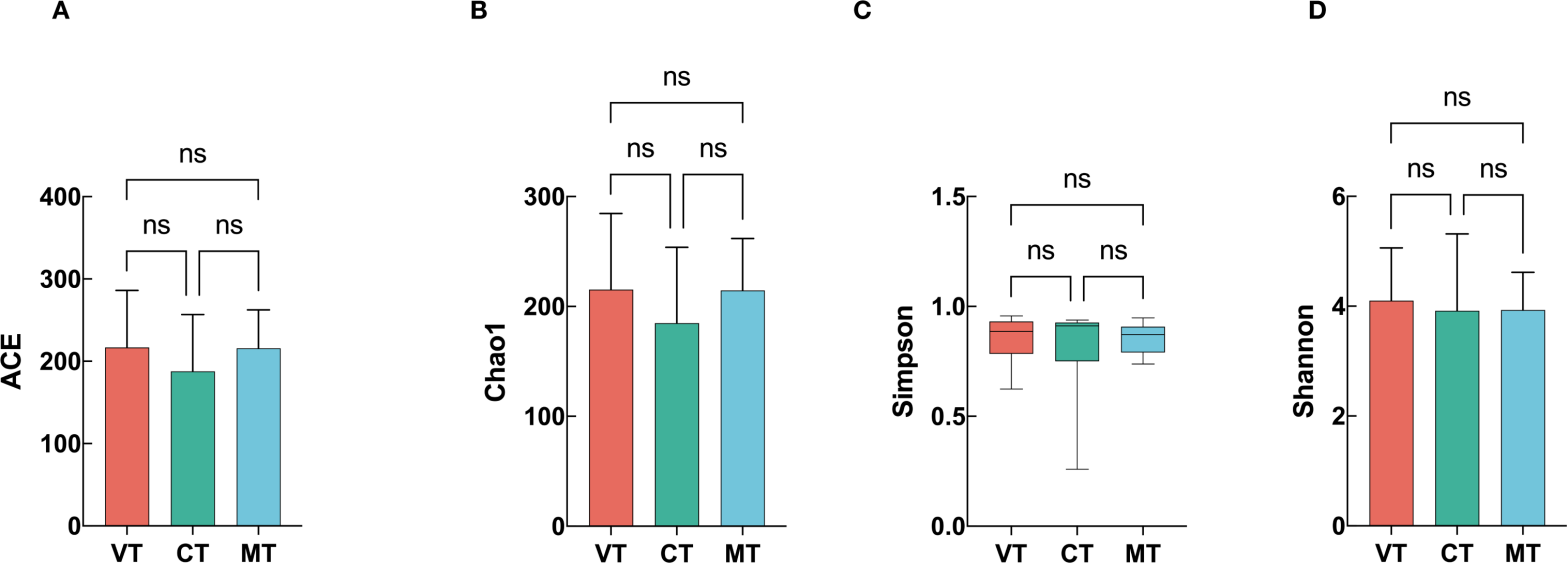
Comparison of gut microbiota alpha diversity indices among the VT, CT, and MT groups. **(A)** ACE index; **(B)** Chao1 index; **(C)** Simpson index; **(D)** Shannon index.

To explore whether differences in gut microbiota existed among VVS subtypes, a preliminary analysis was conducted. The subgroups included the vasodepressor type (VT), cardioinhibitory type (CT), and mixed type (MT). The comparison of alpha diversity indices among the three subgroups is shown below: For the ACE index, the values were 216.770 ± 69.311 in the VT group, 187.881 ± 68.836 in the CT group, and 215.930 ± 46.692 in the MT group, showing no statistically significant difference (*P* = 0.527) (**Figure 3A**). For the Chao1 index, the values were 215.396 ± 69.330 in the VT group, 184.884 ± 68.973 in the CT group, and 214.576 ± 47.257 in the MT group, with no significant difference (*P* = 0.491) (**Figure 3B**). Similarly, comparison of the Simpson index showed values of 0.886 [0.784, 0.932] for VT, 0.912 [0.751, 0.927] for CT, and 0.872 [0.791, 0.908] for MT, with no significant difference (*P* = 0.715) (**Figure 3C**). For the Shannon index, the values were 4.105 ± 0.959 in VT, 3.919 ± 1.401 in CT, and 3.931 ± 0.688 in MT, again indicating no statistically significant difference (*P* = 0.851) (**Figure 3D**). These results suggest that alpha diversity indices did not differ significantly among the three VVS subtypes.

### 3.4 Beta diversity analysis

Beta diversity indices are used to quantify the dissimilarity or similarity of community structures. The Bray-Curtis distance matrix, based on species abundance data, calculates the similarity between samples. And the Weighted UniFrac distance matrix not only incorporates species abundance but also accounts for the phylogenetic relationships between species.

#### 3.4.1 VVS group vs HC group

**Figure 4:**
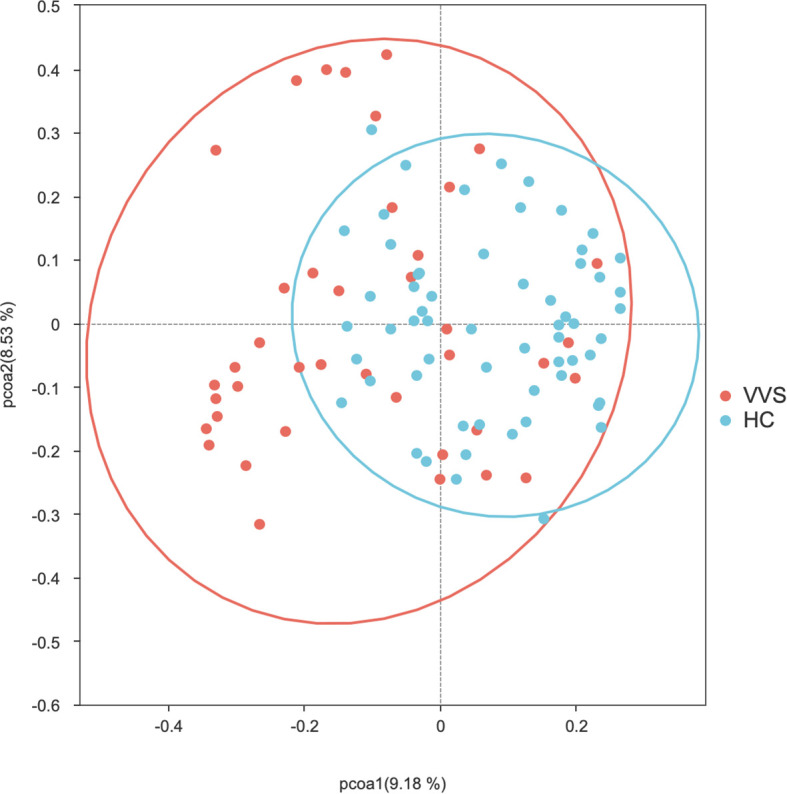
PCoA analysis of the Bray-Curtis distance matrix of the gut microbiota between the VVS and HC groups.

**Figure 5:**
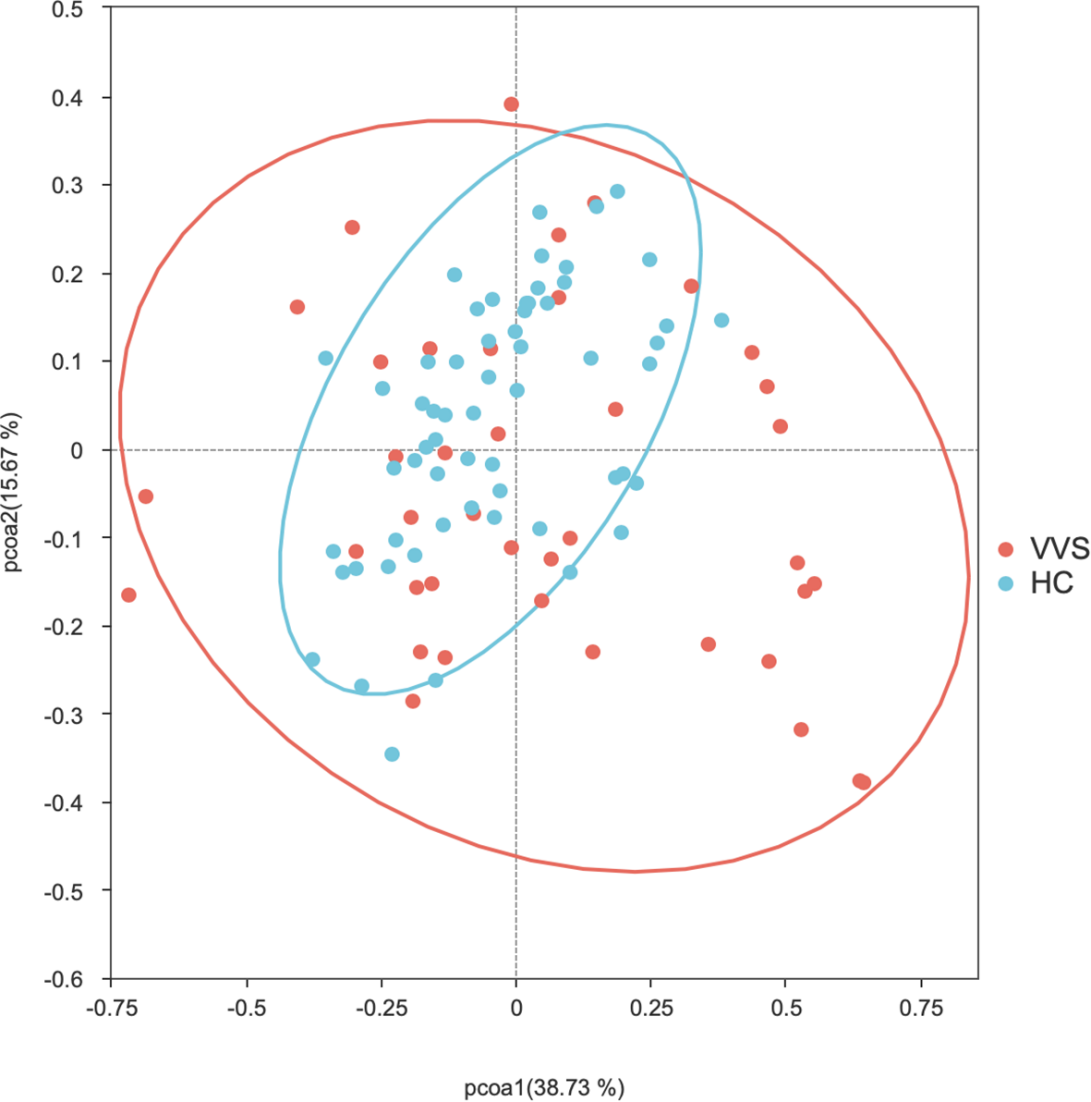
PCoA analysis of the Weighted UniFrac distance matrix of the gut microbiota between the VVS and HC groups.

**Figure 6:**
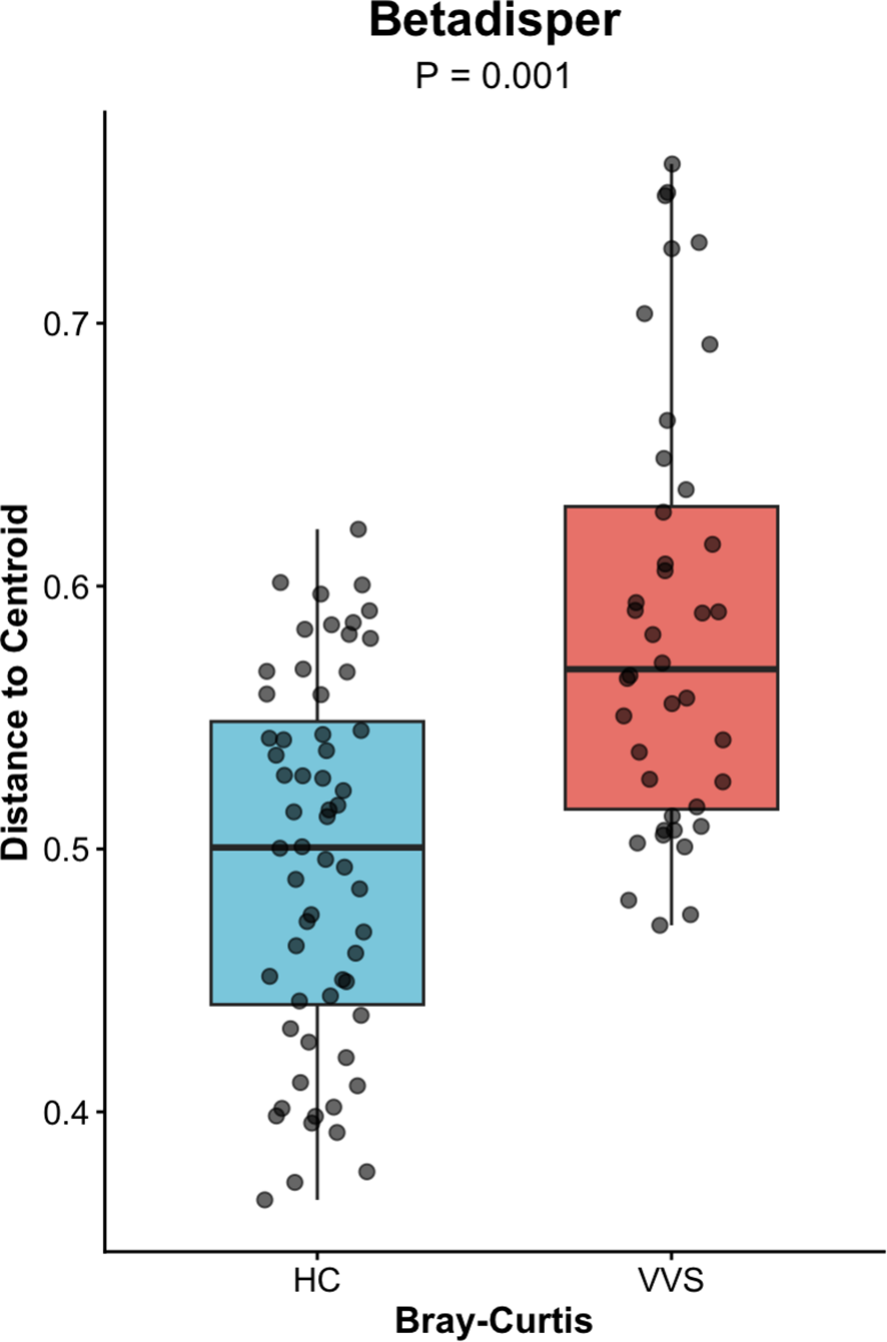
Betadisper analysis of the Bray-Curtis distance matrix of the gut microbiota between the VVS and HC groups.

**Figure 7:**
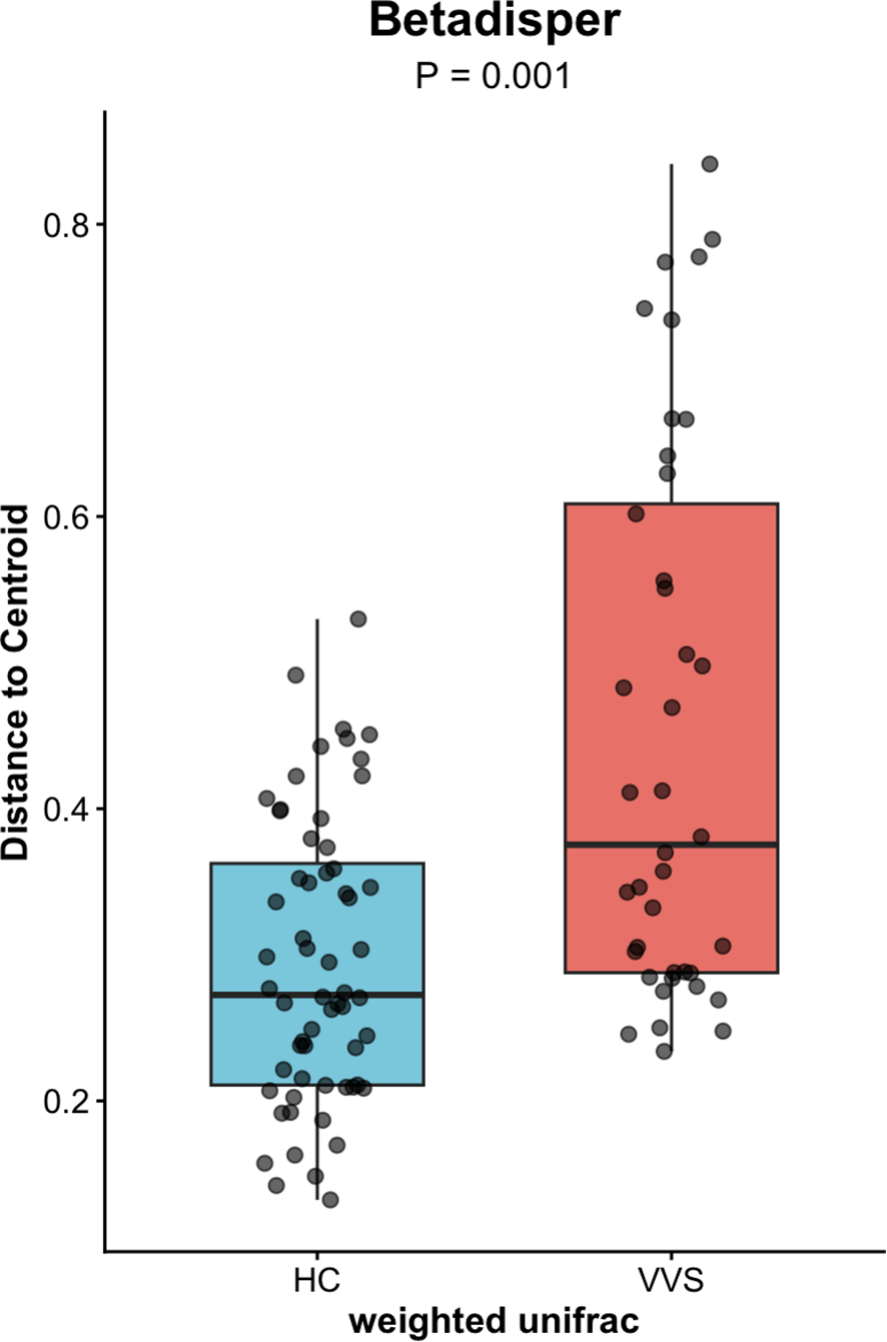
Betadisper analysis of the Weighted UniFrac distance matrix of the gut microbiota between the VVS and HC groups.

PCoA analysis were performed using both distance matrices to compare the differences in gut microbial community composition between the VVS and HC groups. The Adonis test was applied to assess the significance of differences in beta diversity. The microbial community structures of the VVS and HC groups showed modest shift in both Bray-Curtis (*R^2^* = 0.038, *P* = 0.001)* (**Figure 4**) and Weighted UniFrac (*R^2^* = 0.038, *P* = 0.006) (**Figure 5**) PCoA analyses. To further evaluate whether these differences were influenced by heterogeneity of multivariate dispersions, permutational analysis of multivariate dispersions (PERMDISP) was conducted. Correspondingly, boxplots of distances to group centroids were generated to quantify and visualize these variations. The results indicated that the dispersions of beta diversity differed significantly between the VVS and HC groups for both the Bray–Curtis distance matrix (*F* = 34.661, *P* = 0.001) (**Figure 6**) and the weighted UniFrac distance matrix (*F* = 29.206, *P* = 0.001) (**Figure 7**).

In summary, although PERMANOVA revealed a significant shift in microbial community structure between the two groups (*P* < 0.05), the effect size remained relatively modest (*R^2^* = 0.038). This distinction was accompanied by a significant difference in beta-dispersion (*P* < 0.05). Correspondingly, PCoA visualization and dispersion analysis indicated that the VVS group exhibited significantly greater within-group dispersion compared with the control group.

#### 3.4.2 VT group vs CT group vs MT group

**Figure 8:**
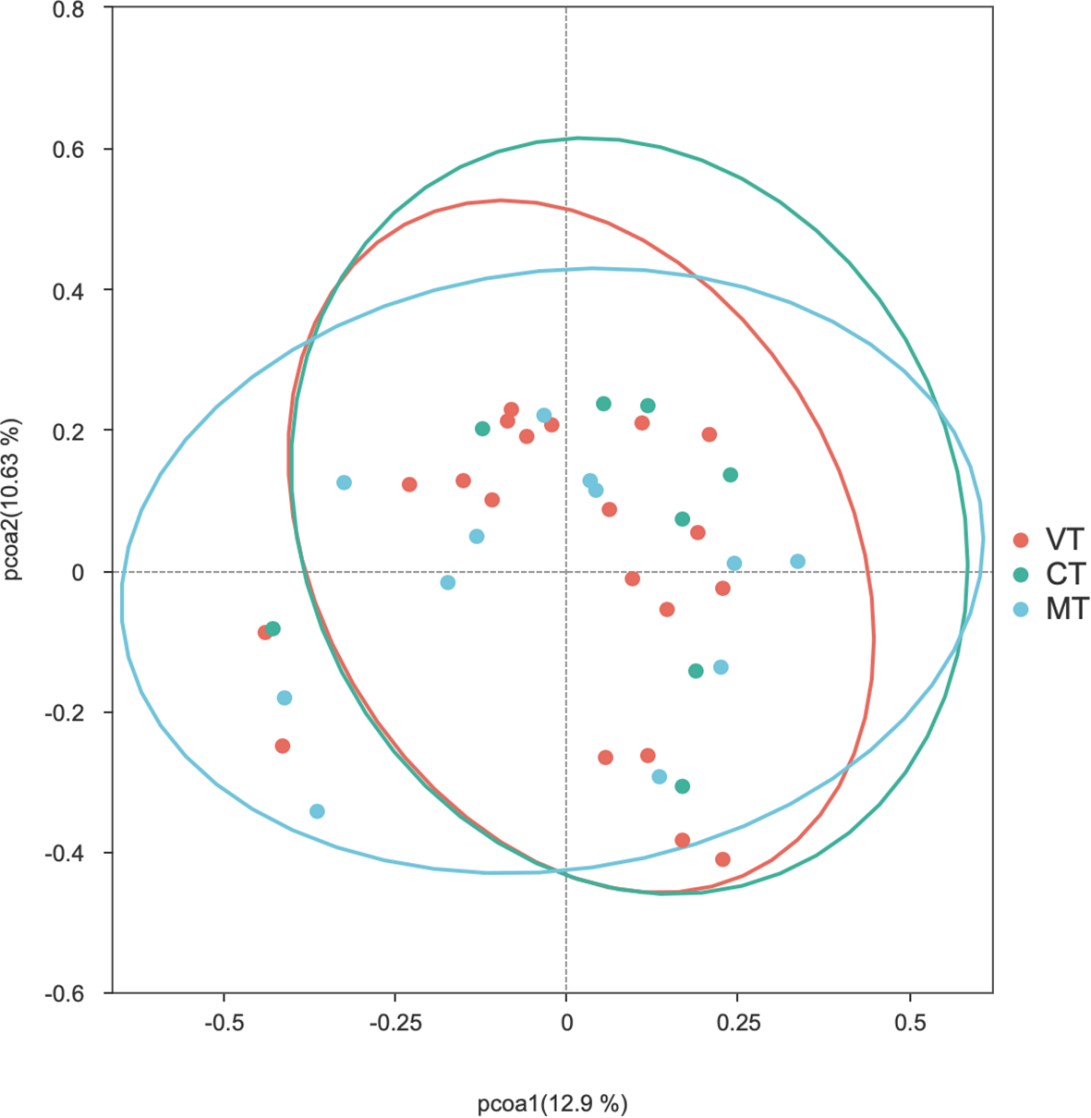
PCoA analysis of the Bray-Curtis distance matrix of the gut microbiota among the VT, CT, and MT groups.

**Figure 9:**
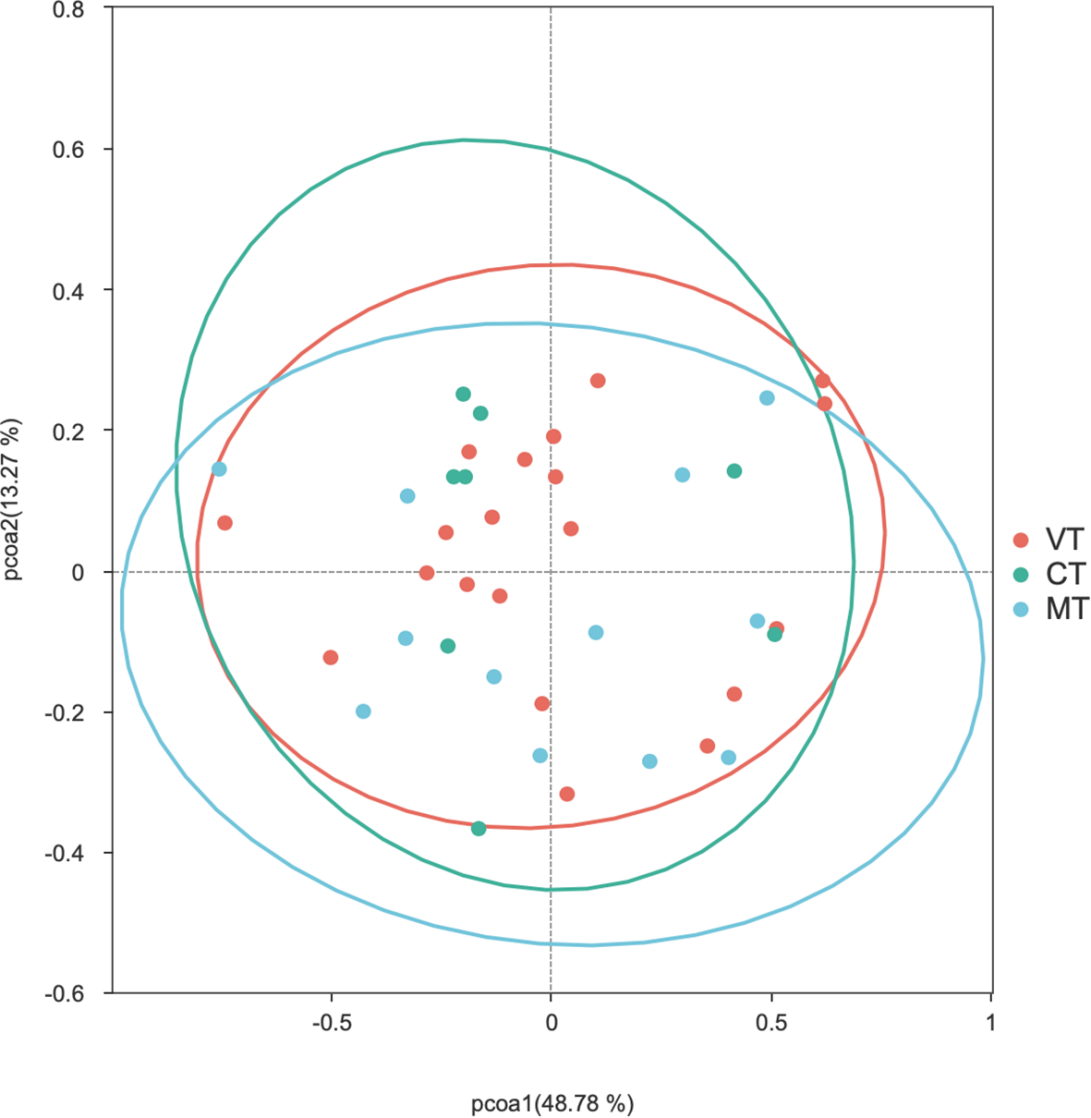
PCoA analysis of the Weighted UniFrac distance matrix of the gut microbiota among the VT, CT, and MT groups.

PCoA analysis was performed using two distance matrices to compare the gut microbiota composition between the VT, CT, and MT groups. Adonis test was applied to assess the significance of Beta diversity. The results indicated that there were no statistically significant differences in the gut microbiota structure among the VT, CT, and MT groups based on both the Bray-Curtis (*P* > 0.05) (**Figure 8**) and Weighted UniFrac (*P* > 0.05) (**Figure 9**) PCoA analyses.

### 3.5 Differences in microbial abundances between VVS and HC groups

**Figure 10:**
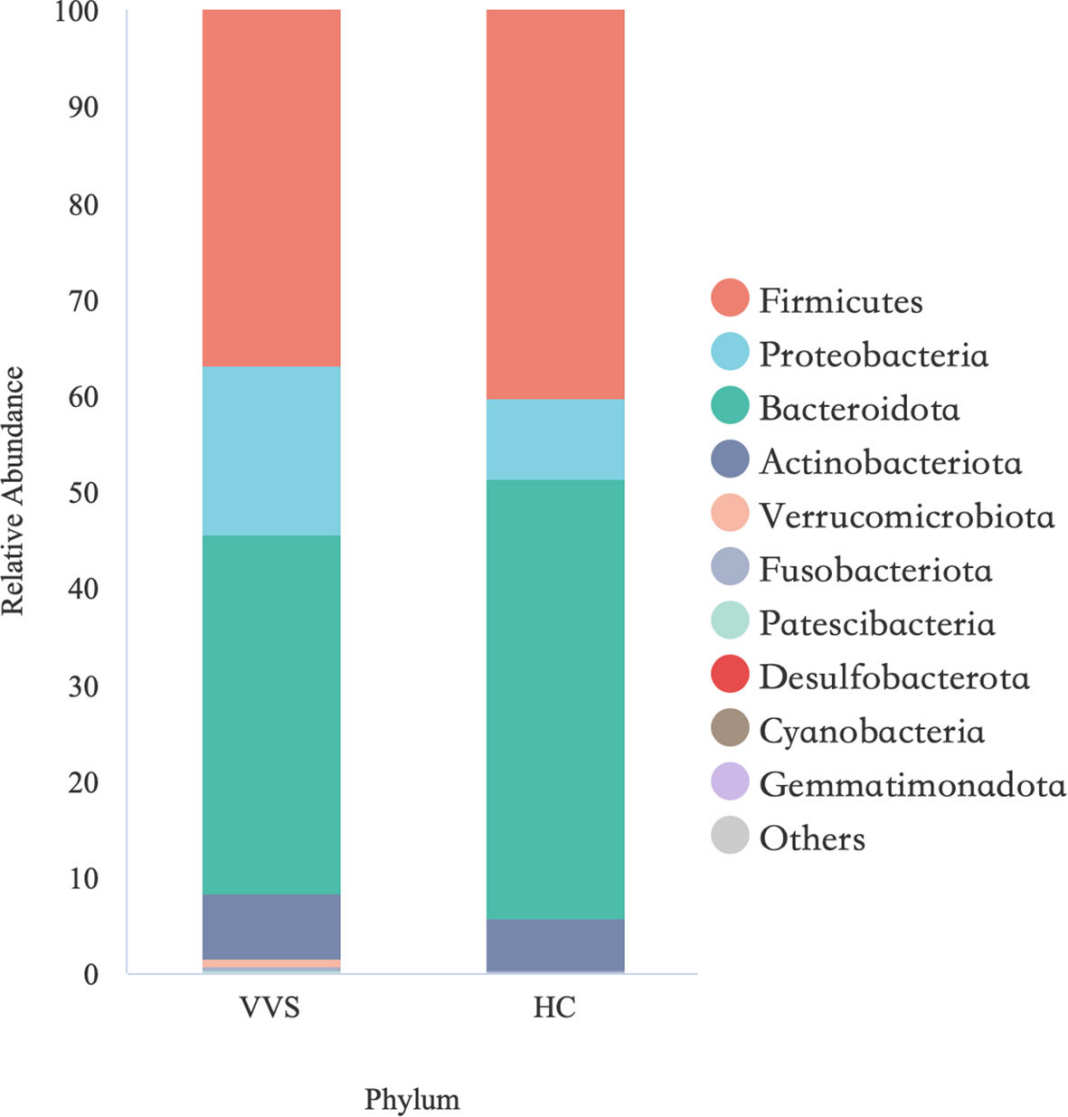
Bar chart showing the top 10 phyla based on relative abundance in VVS and HC groups.

**Figure 11:**
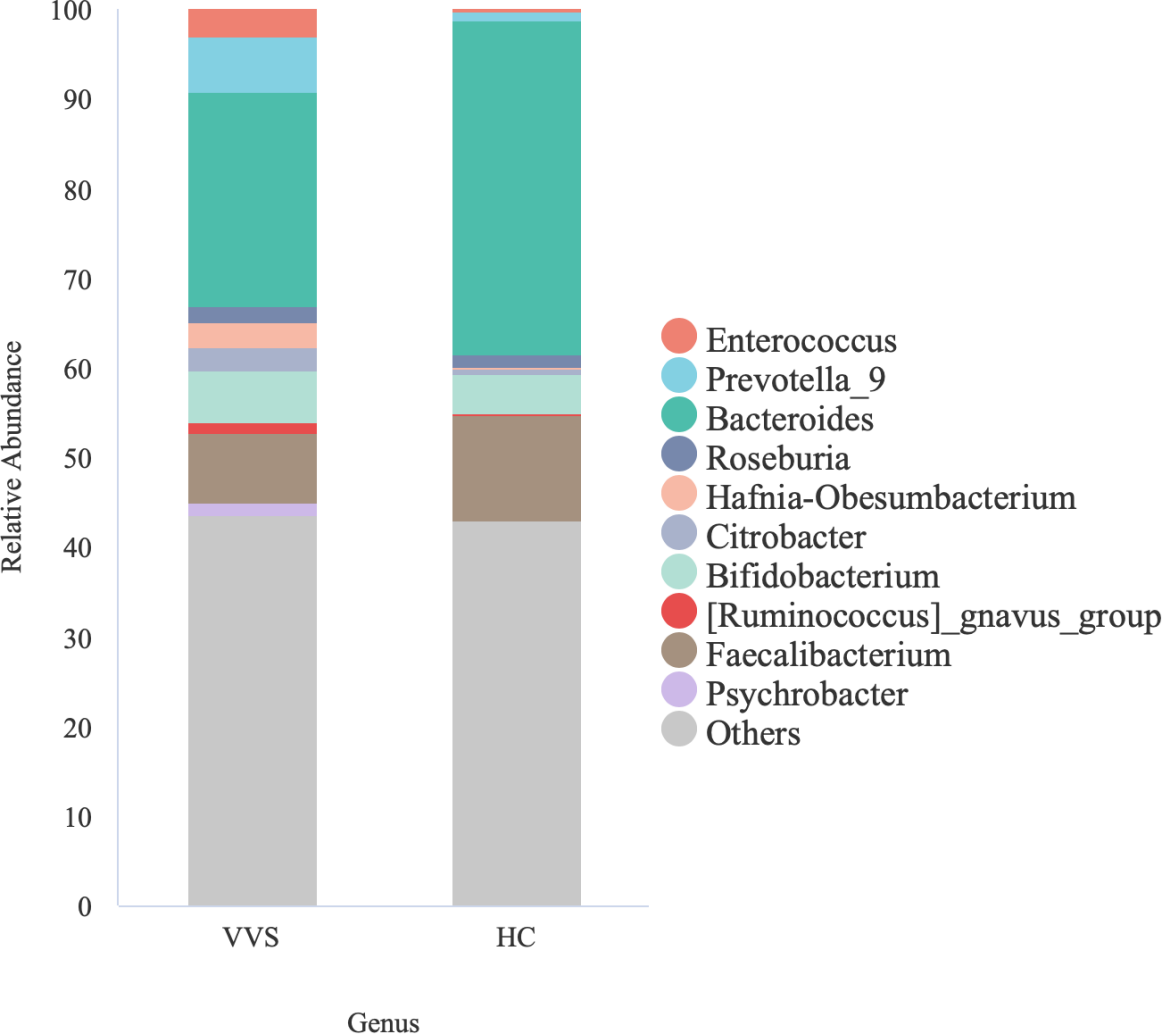
Bar chart showing the top 10 genera based on relative abundance in VVS and HC groups.

Despite the absence of significant differences in gut microbiota among the different VVS subtypes, the microbial alpha diversity in children with VVS was reduced compared with that of HCs, and the beta diversity analyses suggested a tendency toward differences in their microbial community structure. To further explore this, the relative abundance of bacterial taxa in the VVS and HC groups was calculated and visualized using bar plots. At the phylum level, the gut microbiota of the VVS group was mainly composed of Bacteroidota (37.27%), Firmicutes (36.91%), Proteobacteria (17.39%), and Actinobacteriota (6.79%), while that of the HC group was primarily composed of Bacteroidota (45.75%), Firmicutes (40.26%), Proteobacteria (8.31%), and Actinobacteriota (5.18%) (**Figure 10**). The Wilcoxon rank-sum test was employed to assess the differences in phylum-level abundance, with q-values adjusted for multiple comparisons using FDR. No statistically significant differences were observed in the relative abundance of the major phyla between the two groups (*q* > 0.05 **Supplementary Table S1**). At the genus level, we descriptively characterized the dominant bacterial with a relative abundance greater than 5%. In the VVS group, the predominant genera were *Bacteroides* (23.93%), *Faecalibacterium* (7.82%), *Prevotella_9* (6.17%) and *Bifidobacterium* (5.72%), In the HC group, the dominant genera included *Bacteroides* (37.30%), *Faecalibacterium* (11.80%), among others (**Figure 11**).

**Figure 12:**
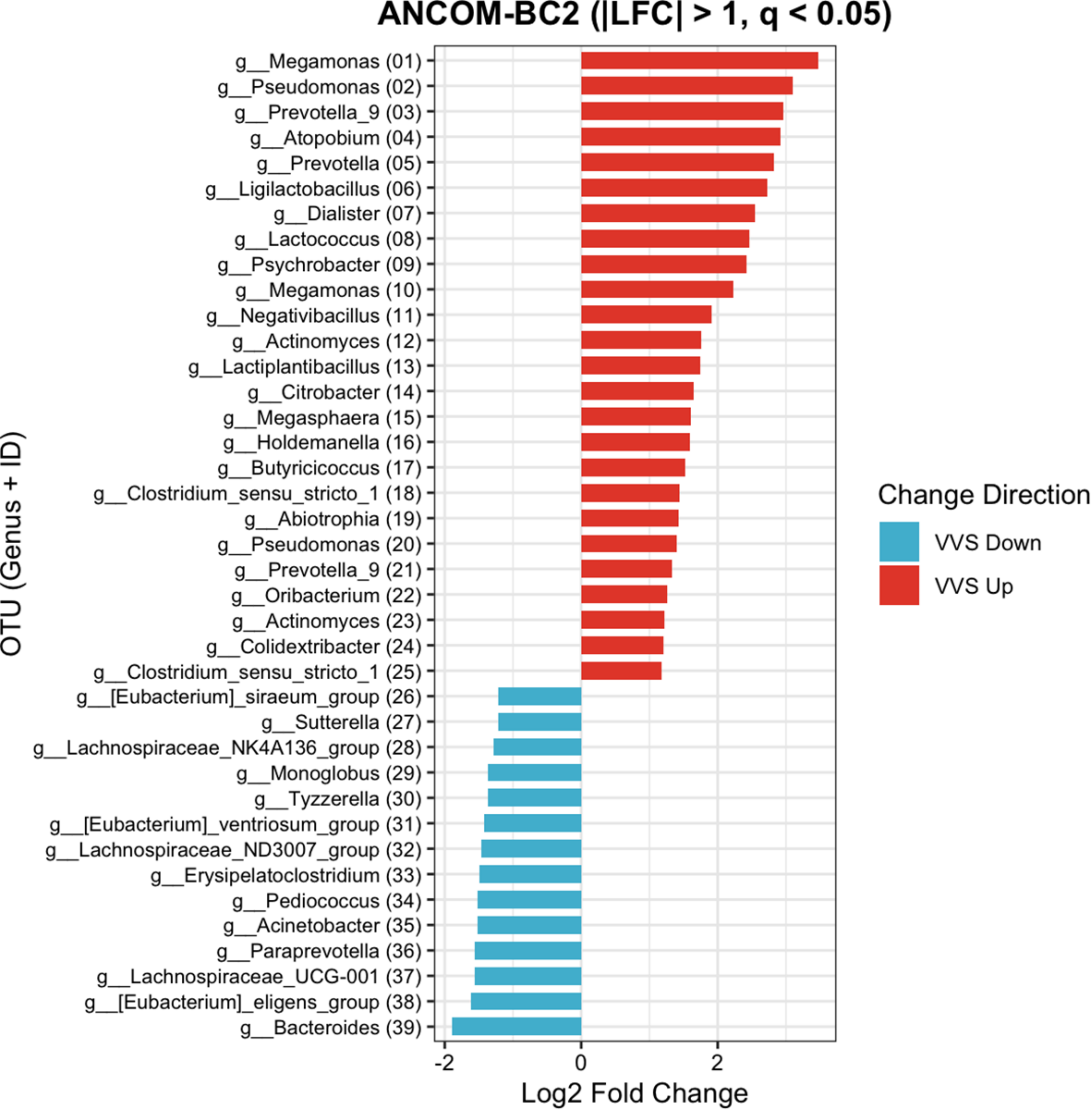
ANCOM-BC2 analysis at genus level between VVS and HC groups. For improved biological interpretability, unclassified and “Others” taxa were excluded from this visualization. See **Supplementary Figure S1** for the complete OTU list.

To identify differentially abundant genera, differential abundance analysis was first performed at the OTU level using ANCOM-BC2, and the resulting differential OTUs with an absolute log fold change (*|*LFC*|*) greater than 1 were subsequently annotated to the genus level. A total of 47 differentially abundant OTUs were identified using ANCOM-BC2. For clarity and biological interpretability, OTUs unclassified at the genus level or annotated as “Others” were excluded from the primary visualization. After filtering, 39 representative OTUs corresponding to 36 distinct genera were retained and displayed in the differential abundance bar plot (**Figure 12**) (The complete list of all identified OTUs is provided in the **Supplementary Material**, **Supplementary Figure S1**). When ranked by log fold change, *Bacteroides* showed the greatest decrease in children with VVS (LFC = −1.89, *SE* = 0.44, *W* = −4.29, *q* = 0.016). In contrast, among the genera enriched in children with VVS, the predominant dominant genus was *Prevotella_9*, which was represented by two differential OTUs showing significant increases (LFC = 2.96, *SE* = 0.50, *W* = 6.45, *q* < 0.001; and LFC = 1.33, *SE* = 0.24, *W* = 5.55, *q* = 0.016).

### 3.6 LEfSe analysis

**Figure 13:**
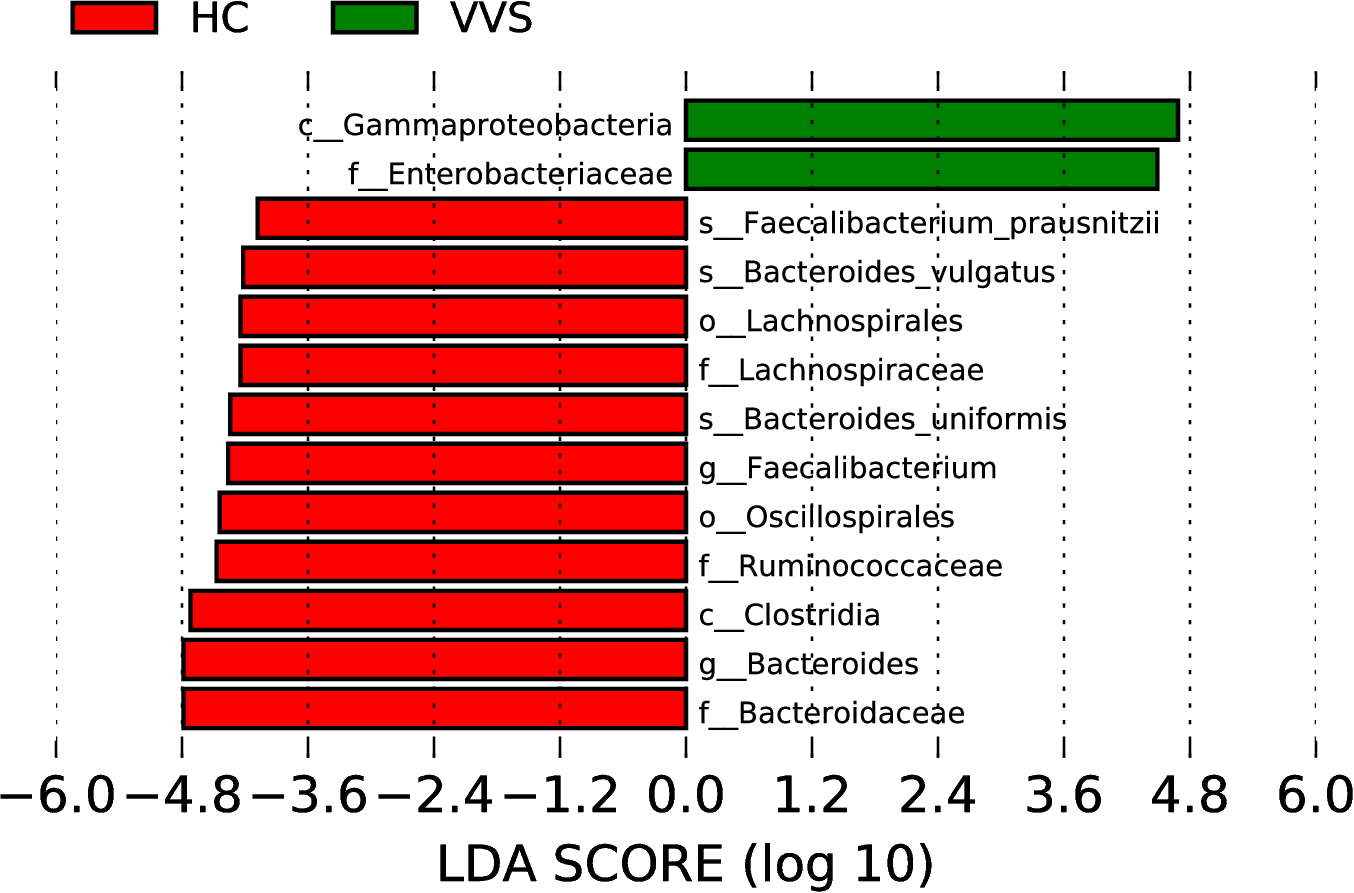
LEfSe analysis of differential species.

LEfSe analysis (LDA score > 4.0, *P* < 0.05) was further applied as a supplementary exploratory analysis to examine differences in microbial community structure, and the results revealed that, at the genus level, *Bacteroides* and *Faecalibacterium* were significantly enriched in healthy children, whereas their abundance was reduced in children with VVS. Both ANCOM-BC2 and LEfSe analyses consistently indicated a significant reduction of *Bacteroides* in children with VVS. In addition, at other taxonomic levels, the abundance of gut microbiota in the VVS group was also reduced, including members of Lachnospiraceae, Oscillospirales, Ruminococcaceae, and Clostridia. (**Figure 13**).

## 4 Discussion

Syncope is one of the most common pediatric emergencies, accounting for approximately 2% of emergency department visits (Wang et al., 2018). VVS is the most prevalent, often characterized by a high recurrence rate and limited response to pharmacological treatment. However, recurrent episodes of syncope can severely affect the physical and mental health, learning ability, and quality of life of affected children. Therefore, elucidating the underlying mechanisms of VVS and optimizing its clinical diagnosis and therapeutic strategies are of great importance. In recent years, advances in high-throughput sequencing and microbiome research have greatly enhanced our understanding of gut microbiota dysbiosis–related disease mechanisms (Shi et al., 2017; Tie et al., 2023). Increasing evidence has demonstrated that gut microbiota imbalance is closely associated with diseases mediated by ANS dysfunction (Naveed et al., 2021), Given that the onset of VVS is tightly linked to ANS regulation, the analysis of gut microbiota in children with VVS may help reveal potential associations between microbial characteristics and disease pathogenesis. Based on this rationale, the present study included 40 pediatric patients with VVS and 60 age- and sex-matched healthy controls. Fecal samples were collected for 16S rRNA gene sequencing to investigate alterations in gut microbial composition among children with VVS and its subtypes. The study revealed the following findings: (1) Children with VVS exhibited reduced alpha diversity, reflected by lower Simpson and Shannon indices, while beta diversity analysis suggested a modest shift in microbial community structure based on Bray–Curtis and weighted UniFrac distances, accompanied by increased within-group dispersion in the VVS group. (2) Among the predominant genera, ANCOM-BC2 identified a decreased abundance of *Bacteroides* and an increased abundance of *Prevotella_9* in children with VVS, while LEfSe analysis revealed reduced abundances of *Bacteroides* and *Faecalibacterium* in the VVS group. (3) No significant differences in gut microbial diversity were observed among the VVS subtypes, including vasodepressor, cardioinhibitory, and mixed types.

Gut microbial diversity is an important indicator of the stability of the microbial community and is generally evaluated by both alpha diversity and beta diversity. Alpha diversity reflects the internal structural characteristics of a community. Specifically, the ACE and Chao1 indices represent community richness and are positively correlated with the number of species, whereas the Simpson and Shannon indices integrate both richness and evenness, providing a comprehensive measure of overall community diversity (Qian et al., 2020). The results showed that there were no significant differences in ACE and Chao1 indices between children with VVS and healthy controls, However, both the Simpson and Shannon indices were lower in the VVS group than in the HC group, suggesting that while species richness was not markedly altered, species evenness was reduced in children with VVS. The combined trends in the Simpson and Shannon indices indicate that the relative abundance of dominant taxa may have decreased in VVS patients, consistent with the findings from the differential taxa analysis. Beyond VVS, reduced alpha diversity of gut microbiota has also been observed in multiple systemic diseases, including irritable bowel syndrome (Zhao and Zou, 2023)、hypertension (Xia et al., 2021)、and bipolar disorder (McGuinness et al., 2022) and so on, indicating that diminished microbial diversity may represent a common feature across various pathological states rather than a disease-specific phenomenon. Such alterations may disrupt the homeostasis of the gut microecosystem, leading to the overgrowth of potentially pathogenic bacteria and the reduction of beneficial commensals, thereby further promoting disease onset and progression. Furthermore, in the alpha diversity analysis among the three VVS subtypes—vasodepressor type (VT), cardioinhibitory type (CT), and mixed type (MT)—no significant differences were observed, suggesting that the microbial distribution patterns were similar among subtypes. However, given the small sample size of the CT and MT groups, the statistical power to detect differences was limited. And further beta diversity analysis is warranted for confirmation.

Beta diversity reflects the degree of dissimilarity in microbial community composition among different samples, representing the heterogeneity of community structures across distinct samples or disease states (Walters and Martiny, 2020). In clinical microbiome studies, beta diversity is commonly visualized using Bray–Curtis and weighted UniFrac distance matrices. The Bray–Curtis distance is calculated based on differences in species abundance, whereas the weighted UniFrac distance incorporates both species abundance and phylogenetic relationships.

In this study, modest but significant shift in microbial community structures were observed between children with VVS and healthy controls under both Bray–Curtis and weighted UniFrac distance metrics. Adonis (PERMANOVA) tests further confirmed that these differences were statistically significant. The concordant results derived from the two distance metrics support the robustness of the findings. However, it is noteworthy that the significant PERMANOVA was accompanied by a relatively modest effect size (*R^2^* = 0.038) and a significant PERMDISP result (*P* < 0.05). Combined with the PCoA and distance-to-centroid boxplot results, these findings suggest that the observed community differentiation was probably driven by increased inter-individual heterogeneity within the VVS group. Such a pattern implies that VVS may destabilize the gut homeostatic state, leading to a more stochastic and individualized microbial configuration—a phenomenon often observed in disease-associated ecosystems. Otherwise, no statistically significant differences in beta diversity were observed among the VVS subtypes. Taken together, these results indicate that children with VVS exhibited noticeable perturbations in gut microbial diversity, characterized by increased inter-individual variation compared to healthy children. Whereas no significant differences in microbial diversity were detected among the different VVS subtypes, however, similar to the alpha diversity analysis, this finding should be interpreted with caution given the limited statistical power.

Building on the significant alterations in gut microbial diversity observed in children with VVS, this study further integrated microbial abundance data with Wilcoxon rank-sum test, ANCOM-BC2, and LEfSe analyses to screen for significant differences between VVS and healthy children. At the phylum level, microbial community composition provides a broad overview of community structure and is relatively stable, serving as a useful indicator for assessing overall gut stability (Orsi and Inagaki, 2023); In contrast, at the genus level, 16S rRNA sequencing offers higher resolution, and bacterial taxa at this level reflect more specific biological functions, making it crucial for understanding the potential roles of specific genera in disease (Michels et al., 2022). Therefore, this study analyzed the differences in microbial abundance in VVS patients at both the phylum and genus levels. The results showed that at the phylum level, the major phyla in both the VVS and healthy control groups were similar, including Bacteroidota, Firmicutes, Proteobacteria, and Actinobacteriota. Previous studies have indicated that the gut microbiota composition at the phylum level remains relatively stable in healthy individuals, predominantly consisting of these four phyla, with Bacteroidota and Firmicutes accounting for over 50%-70% of the total microbial population (Aggarwal et al., 2023). Similarly, in various disease states, the same four phyla have been reported as the predominant components of the gut microbiota (Hu et al., 2022), which is consistent with the findings of this study. This suggests that the gut microbiota exhibits a certain level of stability at higher taxonomic levels, which may help mitigate disruptions caused by environmental differences or disease states, maintaining relatively stable ecological and physiological functions. However, this does not imply that the gut microbiota in children with VVS is identical to that of healthy individuals. As the taxonomic level becomes more refined, the composition and potential functions of the microbiota may exhibit more sensitive or specific alterations (Qiu et al., 2022). At the genus level, significant changes were observed in the major genera of VVS patients compared to healthy children. Based on the ANCOM-BC2 analysis, a total of 36 differentially abundant genera were identified, the majority of which were low-abundance taxa. Given the limited biomass of low-abundance genera within the overall microbial community, our discussion primarily focused on genera with a mean relative abundance exceeding 5%. Among these predominant taxa, *Bacteroides* exhibited the most pronounced decrease in children with VVS, whereas *Prevotella_9* was the dominant genus enriched in the VVS group. Owing to their higher relative abundance, these predominant taxa are more likely to exert substantial biological effects on the host’s physiological state through their collective metabolic output. In contrast, the remaining statistically significant but low-abundance genera likely represent the “rare biosphere” of the gut microbiota. Given their minimal biomass, the potential contribution of these taxa to the pathophysiology of VVS remains unclear and warrants further investigation. These findings suggest an imbalance in the gut microbiota at the genus level in children with VVS, and the abnormal alterations in characteristic genera may be associated with the disease state. Previous studies on the gut microbiota in VVS have reported an increase in the abundance of Ruminococcaceae in VVS patients (Bai et al., 2019), Although this result differs from our findings, it does not imply a direct contradiction between the studies. On the contrary, such differences highlight the potentially complex interactions between the gut microbiota and disease. The microbial community itself is highly complex and dynamic, influenced by various factors such as disease severity, geographical distribution, and sample size differences. Therefore, the divergent conclusions drawn from different studies may reflect the fact that the relationship between gut microbiota and disease is not a simple, linear one, but rather a dynamic and heterogeneous network. In a systematic meta-analysis on bipolar disorder, McGuinness found that most studies observed a reduction in *Faecalibacterium* abundance, while a small number reported an increase (McGuinness et al., 2022). This phenomenon is also observed in other diseases, suggesting that the relationship between the microbiota and disease may be influenced by multiple dimensions and factors. Therefore, the differences observed across various studies should be compared in a more comprehensive and systematic manner, providing clues for the further development of a more unified and comprehensive gut microbiota model. In the case of VVS, there is currently a lack of research on the changes in gut microbiota associated with the disease. Although this study has increased the sample size compared to previous studies, there is still a need for further research in diverse populations to validate these findings and enhance our understanding of the role of the microbiota in the development of VVS.

We observed an increased abundance of *Prevotella_9* in children with VVS. *Prevotella_9* represents a taxonomic subgroup within the genus *Prevotella*, which belongs to the phylum Bacteroidota. Members of the genus *Prevotella* are generally regarded as commensal bacteria and are commonly found in the oral cavity, urogenital tract, and gastrointestinal tract of healthy individuals. However, under conditions of microbial dysbiosis, certain *Prevotella* species may exhibit opportunistic pathogenic potential through the production of various virulence factors (Sharma et al., 2022). A previous study investigating the gut microbiota in children with constipated autism spectrum disorder (C-ASD) reported reduced alpha diversity accompanied by an increased abundance of *Prevotella_9*, a pattern similar to the dysbiotic features observed in the present study (He et al., 2023). Additionally, in a study of type 2 diabetes mellitus (T2DM), the abundance of *Prevotella_9* decreased following therapeutic intervention, indirectly suggesting its potential involvement in disease pathogenesis (Peng et al., 2023). We speculate that in the context of gut dysbiosis across multiple diseases, the bacterial genus consistently shows increased abundance. On one hand, this may be due to the reduction of competing microbiota, and on the other hand, the disease environment itself may facilitate a relationship between *Prevotella_9* and the specific pathophysiological conditions of these diseases.

The significantly reduced *Bacteroides* in children with VVS belongs to the phylum Bacteroidota and is commonly colonized in the gut during infancy (Turroni et al., 2020), making it one of the important symbiotic bacteria in the human gut. Previous studies have reported a decrease in *Bacteroides* abundance in various diseases, including Alzheimer’s disease (Jemimah et al., 2023), hypersensitivity reactions (Hayase et al., 2024), and osteoporosis (Lin et al., 2023), suggesting that this genus may play a critical role in maintaining gut microbiota homeostasis and host health. Fecal lipopolysaccharides (LPS) are components of the outer membrane of the cell wall of Gram-negative bacteria, which are released into the gut contents upon bacterial death or proliferation. Elevated LPS levels promote inflammatory responses that can impair cardiovascular regulation (Liu et al., 2020), Recent studies have found a negative correlation between *Bacteroides* abundance and fecal LPS levels (Yoshida et al., 2020). We speculate that, it is possible that LPS-related processes could be related to the observed microbial alterations. However, these potential associations require further validation through mechanistic investigations.

Another possible explanation may involve short-chain fatty acid (SCFA)–related processes. Both *Bacteroides* and *Faecalibacterium* are major producers of SCFAs in the human gut, primarily in the form of propionate and butyrate (Zafar and Saier, 2021; Wang et al., 2024). The high concentrations of SCFAs in plasma are primarily metabolic byproducts of the gut microbiota (Lal et al., 2001), and therefore, their levels are closely related to microbiota abundance. Notably, a previous study using gnotobiotic mice and an experimental colitis mouse model, revealed a striking ability of Prevotella spp. to perturb the gut microbiome and to decrease the levels of SCFA (Iljazovic et al., 2021). SCFAs have been shown to mediate signaling pathways related to various diseases involving the metabolism, immune system, and nervous system (McKeown et al., 2022; Scheiman et al., 2019; Duscha et al., 2020). A recent study by Huang et al. has demonstrated that propionate promotes axonal regeneration and functional recovery through immune-mediated mechanisms (Serger et al., 2022), and another study by Tomas et al. has confirmed the neuroprotective and regenerative effects of propionate on peripheral nerves (Grüter et al., 2023). Additionally, butyrate also provides a range of protective effects for the host. A study by Huang et al. found that butyrate has potential therapeutic effects in neurodegenerative diseases (Chakraborty et al., 2024). Similarly, Alpino et al. observed that butyrate could exert neuroprotective functions by modulating gene expression in the brain (Alpino et al., 2024).

We speculate that, in the present study, SCFA levels may serve as a potential intermediary linking gut microbiota dysbiosis and ANS dysfunction (Bruning et al., 2020). However, these potential associations require further validation through mechanistic investigations. Regarding the VVS subtypes, no significant differences in microbiota were observed in this study, suggesting that gut microbiota remains relatively stable across different clinical phenotypes of VVS. This indicates that changes in the microbiota may not be a decisive factor for phenotypic differences. On the other hand, this observation may imply that different VVS subtypes share a similar microbiota-mediated pathological mechanism.

This study revealed changes in the gut microbiota of children with VVS, showing a significant reduction in microbiota diversity. These findings provide preliminary theoretical support for gut microbiota intervention in VVS. However, several limitations should be acknowledged. First, although a brief dietary survey was conducted, the enrolled participants exhibited generally similar dietary patterns, the absence of a quantitative dietary assessment may have introduced potential dietary confounding effects on gut microbiota composition. Second, this study used an OTU-based approach with a 97% sequence similarity threshold, which has been widely adopted in previous microbiome studies. Nevertheless, the choice of OTU clustering workflow and parameter settings may influence the analytical results. Therefore, the findings should be interpreted with caution within this methodological context. Third, all participants were Han Chinese from a single province; therefore, the generalizability of the findings to other ethnicities or geographic populations may be limited. In addition, the 16S rRNA sequencing technique used in this study restricts the identification of microbiota at taxonomic levels below the genus level, making it difficult to detect subtle differences between species. Finally, the sample sizes for each subtype were relatively small, particularly for the 8 cases of cardiac inhibition-type VVS and 12 cases of mixed-type VVS. This may affect the statistical power and reproducibility of the results. In the real world, the prevalence of cardiac inhibition-type VVS is lower than that of vasovagal inhibition-type VVS. And children with cardiac inhibition-type VVS may experience conduction block or even cardiac arrest during episodes, which can be misdiagnosed as arrhythmia-related diseases in clinical practice, making it challenging to recruit a larger number of patients.

As an exploratory study, the research aimed to identify potential links between the disease and microbiota. Although the sample size was limited, it still met the exploratory analysis needs of the study. The preliminary results can provide a reference for future multi-center, large-sample studies. In the future, we plan to continue recruiting children with VVS from various subtypes, collect larger sample sizes, explore the associations between differentially abundant gut microbial taxa and clinical indicators of VVS, such as syncope episode frequency, heart rate variability, and blood pressure measurements, and conduct further mechanistic studies using animal models. Additionally, based on gut microbiota research, we look forward to targeted interventions using prebiotics, probiotics, and other therapeutic agents to provide more precise and effective treatments for children with VVS.

## 5 Conclusion

Compared with healthy children, those with VVS exhibited reduced gut microbial alpha diversity and modest compositional alterations. Different analytical approaches identified partially overlapping taxa, reflecting method-dependent variations in differential abundance results. A decrease in *Bacteroides* was consistently observed across analytical methods, suggesting relatively stable findings for this genus and highlighting its potential relevance in VVS. These results indicate a possible association between gut microbiota alterations and autonomic dysfunction. No significant differences in gut microbiota diversity were observed among children with the vasodepressor, cardioinhibitory, and mixed subtypes of VVS.

## Data Availability

The original contributions presented in the study are included in the article/**Supplementary Material**. Additionally, the raw sequence data reported in this paper have been deposited in the Genome Sequence Archive (Genomics, Proteomics & Bioinformatics 2025) in the National Genomics Data Center (Nucleic Acids Res 2026), China National Center for Bioinformation / Beijing Institute of Genomics, Chinese Academy of Sciences (GSA: CRA039810), which are publicly accessible at https://ngdc.cncb.ac.cn/gsa. Further inquiries can be directed to the corresponding author.
