# Supplementary figures and images for: Reduced gut microbial diversity characterized by decreased Bacteroides is associated with vasovagal syncope in children
Source: Front Cell Infect Microbiol. 2026 Apr 14;16:1764992. doi: 10.3389/fcimb.2026.1764992 (PMC13121255; doi:10.3389/fcimb.2026.1764992)

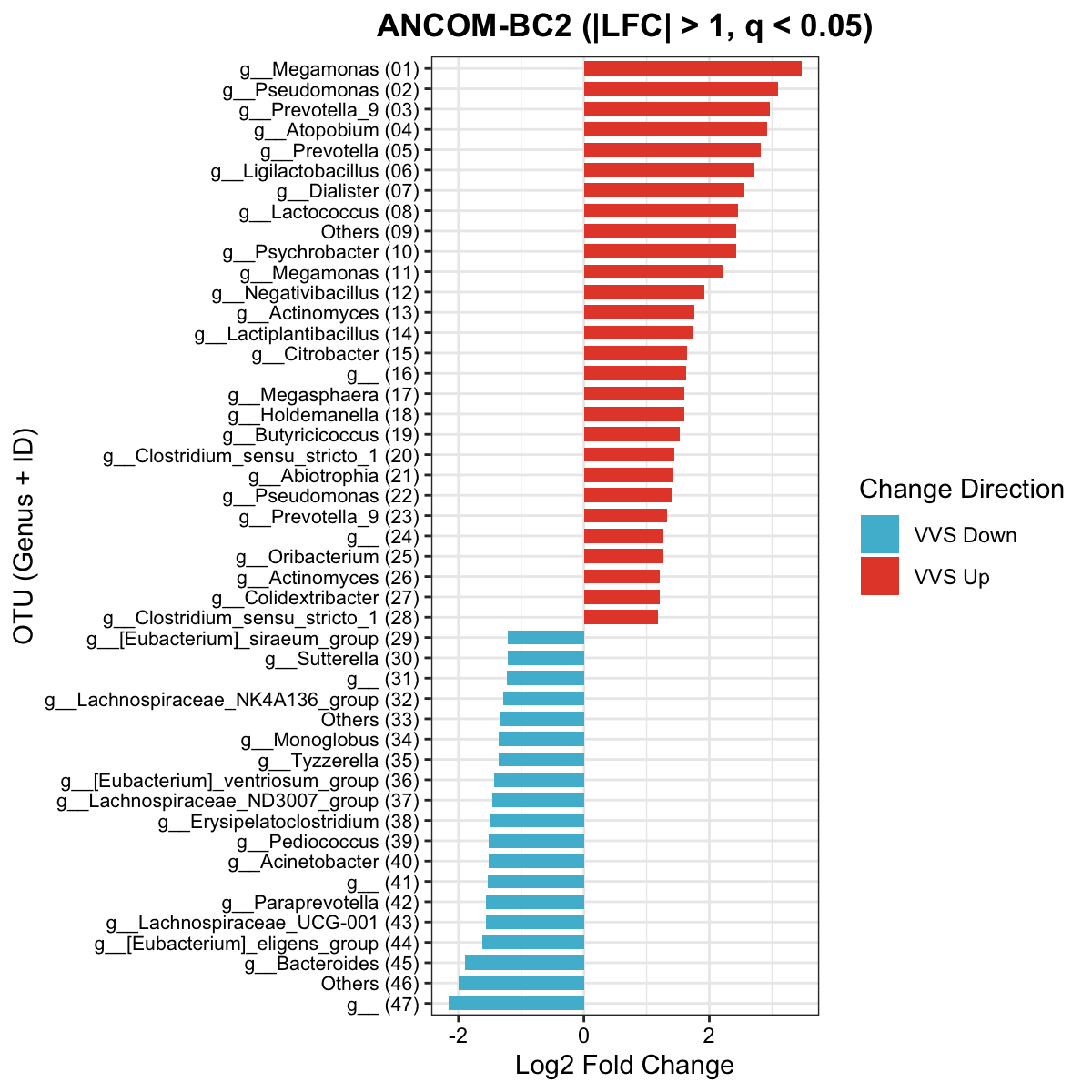

Supplement: Supplementary file 1 [file Image1.tiff]
